# Exploring the interaction and impact of probiotic and commensal bacteria on vitamins, minerals and short chain fatty acids metabolism

**DOI:** 10.1186/s12934-024-02449-3

**Published:** 2024-06-12

**Authors:** Luis G. Bermúdez-Humarán, Benoit Chassaing, Philippe Langella

**Affiliations:** 1 Laboratory of Commensals and Probiotics-Host Interactions, Université Paris-Saclay, INRAE, Micalis Institute, Jouy-en-Josas, AgroParisTech 78350 France; 2 Microbiome-Host Interactions, Institut Pasteur, Université Paris Cité, INSERM U1306, Paris, France; 3 INSERM U1016, team Mucosal microbiota in chronic inflammatory diseases, CNRS UMR 8104, Université de Paris, Paris, France

**Keywords:** Probiotics, Vitamins, Minerals, Energy metabolism, Immunity, Gut health, Synergy

## Abstract

There is increasing evidence that probiotic and commensal bacteria play a role in substrate metabolism, energy harvesting and intestinal homeostasis, and may exert immunomodulatory activities on human health. In addition, recent research suggests that these microorganisms interact with vitamins and minerals, promoting intestinal and metabolic well-being while producing vital microbial metabolites such as short-chain fatty acids (SCFAs). In this regard, there is a flourishing field exploring the intricate dynamics between vitamins, minerals, SCFAs, and commensal/probiotic interactions. In this review, we summarize some of the major hypotheses beyond the mechanisms by which commensals/probiotics impact gut health and their additional effects on the absorption and metabolism of vitamins, minerals, and SCFAs. Our analysis includes comprehensive review of existing evidence from preclinical and clinical studies, with particular focus on the potential interaction between commensals/probiotics and micronutrients. Finally, we highlight knowledge gaps and outline directions for future research in this evolving field.

## Introduction

**Fig. 1:**
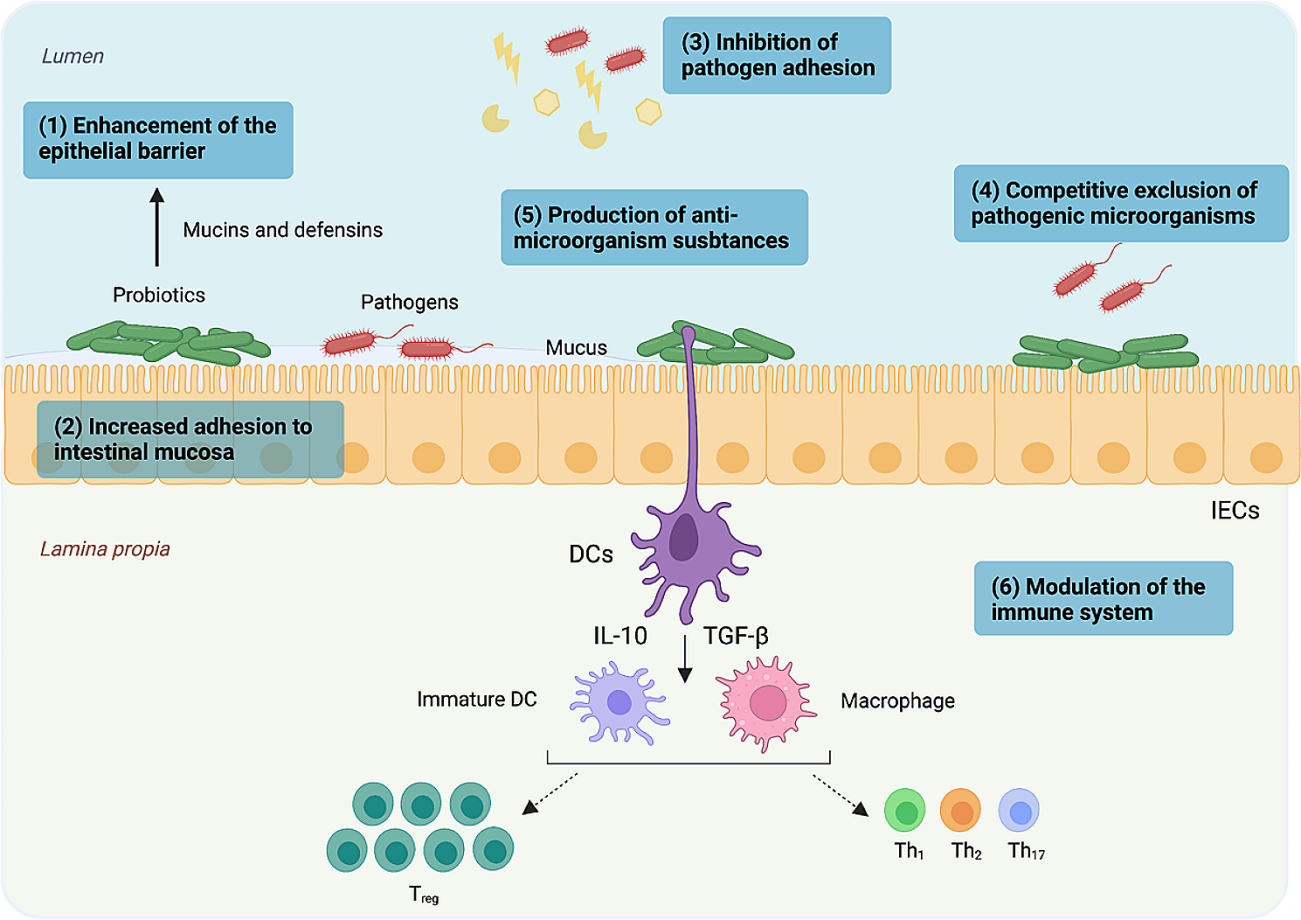
Main probiotics mechanisms of actions. This figure was created with Biorender.com and reproduced with permission from Bermudez-Brito et al. [134])

Probiotics are defined as *“live microorganisms that, when administered in adequate amounts, confer a health benefit on the host”* [1, 2]. The “adequate amount” of a probiotic is strain-dependent and is broadly defined as the quantity proven to provide a health benefit in human clinical trials [3]. Probiotic bacteria exert multiple effects on the intestinal environment, although precise mechanisms of action are not yet fully understood [4, 5]. Thus, probiotics play a key role in substrate metabolization, food digestion and energy recovery [5]. Probiotics also contribute to epithelial barrier integrity through their ability to restore intestinal permeability and decrease inflammation (Fig. 1) [6]. However, not all probiotics display similar properties, and these effects appear bacterial-strain specific [7, 8].

In healthy individuals, the intestinal microbiota and the host immune system exist in a state of homeostasis. This homeostasis can be disrupted, leading to a dysbiotic gut microbiota, which has been observed in many human diseases, such as inflammatory bowel diseases (IBD), obesity, type 2 diabetes, and several types of cancers [9, 10]. In addition, recent data suggest a link between gut microbiota bioenergetics and chronic metabolic diseases, with a deficit of shared energetic resources in the dysbiotic microbiota of patients [11]. Furthermore, a causal correlation between gut bacteria and disease onset (or, conversely, disease protection) has been established at the molecular level [10].

The use of probiotics has demonstrated its beneficial potential beyond a simple modulation of the intestinal microbiota composition. Indeed, probiotics have shown promise in the treatment and management of various diseases. For example, in gastrointestinal disorders such as IBD and irritable bowel syndrome (IBS), they improve the diversity of the gut microbiota, strengthen intestinal barrier, modulate immune responses and produce antimicrobial substances [12, 13]. In metabolic disorders such as obesity and type 2 diabetes, probiotics improve energy metabolism and insulin sensitivity [14], and reduce inflammation through the production of short-chain fatty acids (SCFAs) [15]. In allergic conditions such as eczema and allergic rhinitis, they regulate the immune response and promote a healthy gut microbiota to reduce allergic reactions [16]. Probiotics also help prevent urogenital infections, such as bacterial vaginosis and urinary tract infections, by competing with pathogens and producing antimicrobial compounds [17]. New research suggests that probiotics may have a positive impact on mental health by influencing the gut-brain axis and stress response mechanisms. In addition, in skin disorders such as acne and psoriasis, probiotics reduce inflammation and balance the skin microbiota [18]. These diverse functions highlight the therapeutic potential of probiotics to maintain and restore health in a variety of medical settings.

For instance, beneficial effects of selected strains of *Lactobacillus* and *Bifidobacterium*, the best documented traditional probiotic bacterial genera to date, have been reported both in vitro and in preclinical and clinical trials [7, 19]. Besides, scientific advances in genome sequencing techniques and culture methods have allowed the isolation and characterization of commensal bacterial strains such as *Faecalibacterium prausnitzii*, *Christensenella minuta* and *Akkermansia muciniphila*, with potential health benefits and the opportunity to be developed as next-generation probiotics (NGPs) or live biotherapeutics (LBPs) [19, 20]. Most of these NGPs have been identified by comparing the gut microbiota of healthy versus sick individuals [21]. Today, traditional probiotics are used in functional foods and food supplements, whereas NGPs are used as drug products or, more recently, as a novel food in Europe [22, 23]. Apart from commensals, there is a great interest in a second type of NGPs, which are genetically modified or engineered bacteria, such as *Escherichia coli* Nissle 1917 or lactic acid bacteria (LAB). Over the past decade, the main challenges of using such NGPs, especially related to their delivery and consumption, have been circumvented [24].

Understanding mechanisms underlying probiotic effects within the human gut may contribute to a rational selection of probiotic strains in both healthy and sick individuals [9]. In this context, food-grade microorganisms, such as lactobacilli and bifidobacteria, may play a key role in modulating host micronutrient status through their ability to synthesize vitamins and increase nutrient (e.g. minerals) absorption in the gut [4, 10, 11, 19]. Furthermore, some bacterial strains produce a range of beneficial molecules such as amino acids, enzymes, and SCFAs [25]. Additionally, probiotics may play a role in modulating host immunometabolism through the production of SCFAs and tryptophan (*trp*) metabolites, among others, which altogether impact intestinal inflammatory status and host metabolic health [26]. Indeed, recent studies have identified key proteins involved in the *trp* metabolic pathway, such as Aryl hydrocarbon Receptors (AhR), as targets for the treatment of gastrointestinal diseases, inflammation, and malignancies [27]. Of note, the commensal *Limosilactobacillus reuteri* F6 strain has been successfully used to restore AhR activation in mice [28], and novel NGPs are being developed for their ability to activate AhR [27].

Finally, recent data suggest that probiotics may act synergistically with micronutrients (such as vitamins and minerals) to boost intestinal immunity and promote human health. The modes of action involved in such synergy between the triad probiotics–vitamins–minerals are complex and not yet fully elucidated [29]. In this review, we summarize the main hypotheses regarding mechanisms of action of probiotics in the gut and their add-on effects on the bioavailability and metabolism of vitamins and minerals. We assess current evidence from pre-clinical and clinical studies and provide clues on the synergistic effects of probiotics and micronutrients, as well as highlighting knowledge gaps and needed future research directions. Briefly, we will describe and discuss *i*) the activity of probiotics on host energy metabolism, *ii*) the role of vitamins and minerals in probiotic energy metabolism and finally, *iii*) the synergistic effects of probiotics, vitamins and minerals on human health.

## Probiotics in host energy metabolism

Humans lack the enzymes needed to break down fermentable fibers, which pass unaffected the upper gastrointestinal tract (GIT) and are fermented in the cecum and large intestine by the gut microbiota [30]. Some particular strains of either probiotics or commensal bacteria have the ability to break down indigestible fibers, leading to the production of multiple types of beneficial metabolites, of which SCFAs (mainly acetate, propionate and butyrate) are the most abundant [30–33]. These three SCFAs have different tissue distributions and effects on host physiology [34]: (i) acetate produced by colonic bacteria enters the blood compartment, where it is mixed with endogenous acetate released by tissues and organs [30], and (ii) propionate acts as a precursor for gluconeogenesis in the liver [30, 34] and butyrate is preferentially used as an energy source by colonocytes [30, 34, 35]. It is converted into acetyl-CoA, which enters the cellular Krebs cycle to provide energy in the form of adenosine triphosphate (ATP) [36, 37].

SCFAs also play a central role in regulating host intestinal health through several mechanisms, such promoting the integrity of the intestinal barrier and preventing leaky gut syndrome [38]. SCFAs also have anti-inflammatory properties and regulate the immune response by modulating cytokine production [39]. They influence intestinal motility by stimulating hormones such as peptide YY (PYY) and GLP-1, and help maintain a balanced intestinal microbiota [40]. In addition, SCFAs increase mucin production, potentially reinforcing the protective mucous layer of the intestine. Through epigenetic regulation as histone deacetylase (HDAC) inhibitors, SCFAs affect gene expression and cellular processes [41]. They also interact with G protein-coupled receptors (GPCRs), influencing physiological functions such as immune response and hormone release [42]. These multifaceted actions make SCFAs crucial for intestinal and overall health.

Therefore, probiotic bacteria have emerged as an attractive alternative to modulate host energy balance through their role in SCFAs production [43]. While SCFAs are produced primarily for bacterial needs, they can also cross the intestinal epithelium and be used by the host as a nutrient source [44]. Moreover, probiotic metabolites, including SCFA, act as signaling molecules that impact energy uptake, storage and expenditure, as well as appetite [43]. Additionally, there is a well-established crosstalk between the gut microbiota and the brain (i.e. gut-brain axis), mediated by SCFAs and other metabolites [45, 46]. This crosstalk helps to regulate nutrient signaling and maintain energy homeostasis, and its dysfunction may contribute to obesity [47].

## Vitamins and minerals as key actors in host energy metabolism modulation by probiotics

**Fig. 2:**
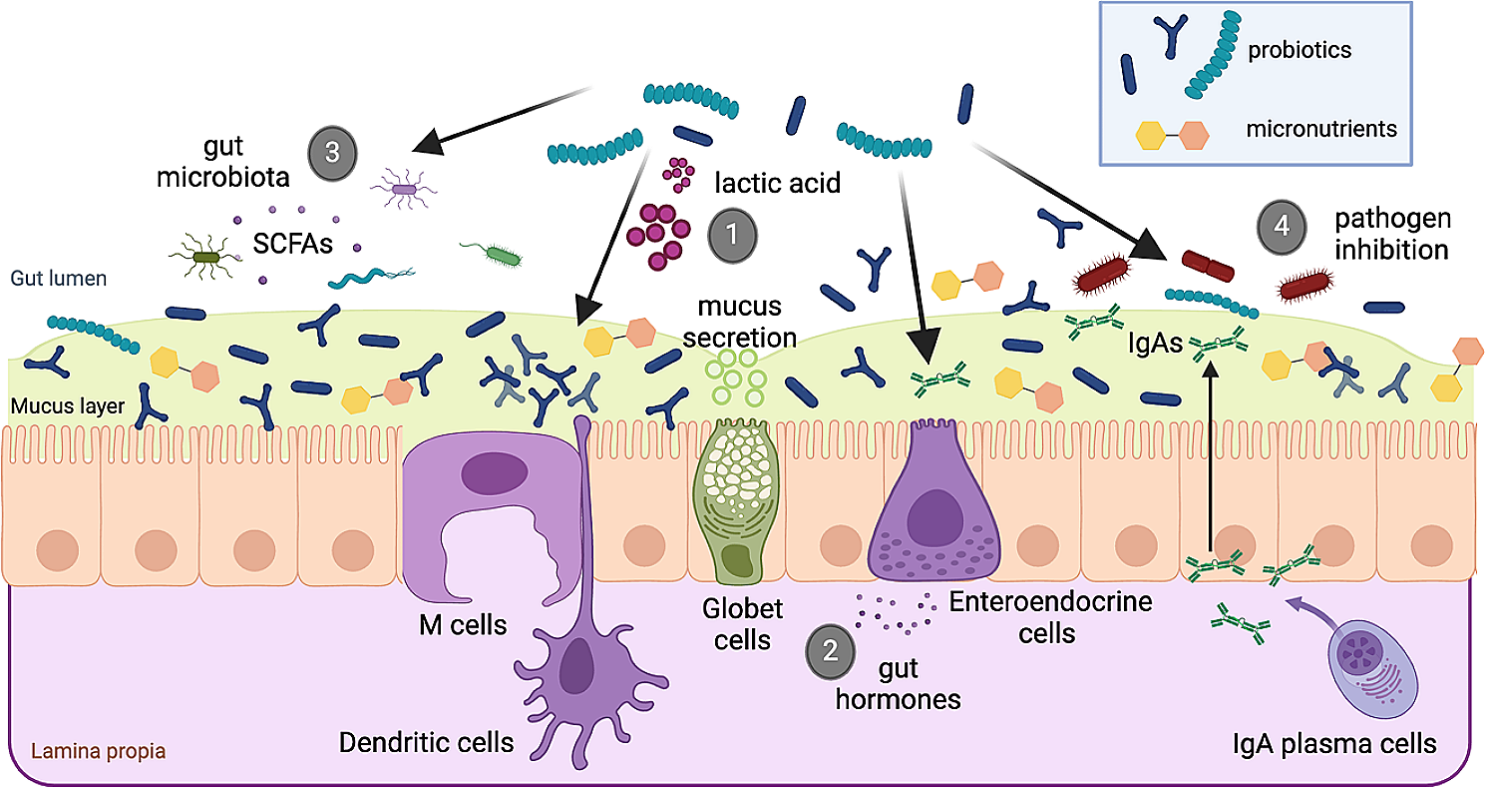
Proposed mechanisms by which probiotics optimize the intestinal environment for better nutrient absorption. This figure was created with Biorender.com (Based on the text in Barkhidarian et al. [29])

While the intestinal microbiota plays a pivotal role in food digestion and energy recovery, it can also produce and supply vitamins to its host [5, 48–51]. Selected probiotics may thus optimize vitamins and minerals absorption in the gut through a number of different mechanisms including: (i) lowering pH through increased production of intraluminal lactic acid, (ii) modulating hormone levels, (iii) beneficial alterations of the gut microbiota, and (iv) inhibiting pathogenic bacteria adhesion to the surface of intestinal epithelial cells, thereby reducing competition for available nutrients (Fig. 2) [29].

B vitamins are water-soluble vitamins required as cofactors for enzymes that catalyze cleavage of energy from nutrients to form ATP [5, 49]. Food-related LAB, as well as human gut commensals, can synthesize *de novo* and supply most B-vitamins, e.g. folates (B9), riboflavin (B2), cobalamin (vitamin B12), thiamine (B1) and pyridoxine (B6) [5, 52, 53]. The genes responsible for vitamin biosynthesis in LAB have been identified in several species [54]. For example, cobalamin has been found to be produced by different strains of *Lactobacillus reuteri* [55]. Vitamin production by LAB varies significantly, as it is a species-specific or strain-dependent trait. However, studies have shown that this property is not bacterial species-specific but rather bacterial strain-specific [56–59]. For instance, some particular strains of LAB or Bifidobacteria have the ability to produce vitamins such as the folate-producing probiotic strains *Bifidobacterium longum* B6 and ATCC 15,708, *Lactobacillus acidophilus* N1 and ATCC 4356, *Lactobacillus delbrueckii* ssp. *bulgaricus* 448 and 449, and *Streptococcus thermophilus* MC and 573 [60], *Lactobacillus sakei* strains CRL 2209 and CRL 2210 [61] and *Bifidobacterium catenulatum* ATCC 27,539 [62]. Also, a study evaluating the in vitro potential of four probiotic bacterial strains to produce and release *de novo* SCFAs and selected B-vitamins found that *B. longum* SP 07/3 and *B. bifidum* MF 20/5 were able to synthetize in vitro thiamine but unable to synthesize folates or riboflavin [52]. The two bifidobacterial strains, together with *Lactobacillus gasseri* PA 16/8, were also able to produce propionate and acetate. The same study suggested that SCFAs- and vitamin B-producing bacteria can optimize ATP production in hosts [52].

It has been hypothesized that, if a bacterial strain is able to synthesize vitamins within the gut, it may thus increase the total vitamins content of the host [48]. Indeed, the ability of selected bacterial strains to boost vitamins absorption has been shown in pre-clinical and clinical studies. For instance, *Bifidobacterium adolescentis* MB 239 was found to increase folate levels [63], while vitamins-producing LAB improved both folate [64] and riboflavin status in vitamins-deficient animals [65, 66]. Moreover, in a randomized, double-blind, placebo-controlled trial of 46 obese patients undergoing gastric bypass, it was observed that probiotic supplementation with 7 bacterial strains (*Lacticaseibacillus casei, L. rhamnosus, Lactobacillus acidophilus, Lactobacillus bulgaricus, Streptococcus thermophilus, Bifidobacterium breve* and *B. longum*) plus prebiotic (fructo-oligosaccharides [FOS]) improved vitamin D3 and B12 levels at month 4 compared to placebo [67]. Prebiotic has been defined as a substrate that is selectively used by host micro-organisms conferring a health benefit [68]). Interestingly, these effects were transient and did not persist at 9-months follow-up, suggesting that long-term probiotic supplementation should be considered [67]. Another randomized trial demonstrated that daily intake of a probiotic capsule containing 2.4 billion of *L. acidophilus* La-14 significantly increased vitamin B12 levels in morbidly obese patients compared to the control group [69].

More recently, a synergy between vitamin D3 and *L. rhamnosus* GG was shown to protect mice from colitis [70] through the promotion of Vitamin D Receptor expression and epithelial cells proliferation. This preclinical study shows that vitamin D3 could synergize with the probiotic strain *L. rhamnosus* GG, providing therapeutic potential in IBD.

Moreover, in the MetaCardis cohort study, biotin level in relation with the gut microbiota was evaluated in 1,545 obese individuals [71]. This study demonstrated that microbiota can lose the ability to generate biotin in subjects with severe obesity. More importantly, in mice models, supplementing high-fat diet-fed mice with FOS and biotin was found to exert a synergistic effect on microbial diversity, biotin and B-vitamin levels, while limiting weight gain and impaired glycemia [71].

Probiotics supplementation can also increase the concentration of minerals in fermented foods [49] and there is evidence that these microorganisms also play a role in mineral absorption [72]. The use of synbiotics, i.e. combinations of probiotics and prebiotics, has also been shown to have an additional effect on mineral absorption [72]. In this context, a number of studies have evaluated the effect of probiotic administration on mineral absorption [73–77]. Thus, recent work concluded that *Lactobacillus* spp. and *Bifidobacterium* spp. have beneficial effects on the bio-accessibility and bio-availability of major minerals including iron, zinc, magnesium, calcium, and selenium [74]. Animal studies found that probiotic supplementation of broiler feeds significantly raised serum calcium and iron levels and improved digestive function and physiological status in chickens [75, 76]. Other studies using prebiotics showed that stimulation of commensal bacterial population levels can increase mineral absorption in animals [78].

In humans, a systematic review of probiotics studies in healthy individuals indicated that probiotic consumption improves calcium levels in pediatric, geriatric and postmenopausal subjects and may facilitate zinc uptake in children. However, results regarding iron absorption were less congruent [29]. While some clinical trials focusing on the effect of probiotic supplementation on zinc and iron deficiency showed conflicting results [79], a randomized pilot study of 40 pediatric patients demonstrated that a selected combination of *Lactiplantibacillus plantarum*, *L. acidophilus*, *Bifidobacterium infantis* and *Bifidobacterium lactis* is effective in increasing the levels of calcium, zinc and iron if administered for at least 5 weeks [80]. In people with type 2 diabetes, supplementation with a mixture of 7 probiotic strains for 8 weeks significantly raised serum calcium concentrations *versus* placebo in a randomized controlled trial (*n =* 58) [73].

Also, in post-menopausal women, a clinical study demonstrated a positive effect was observed following probiotic supplementation, with beneficial effects on bone metabolism and bone mass density in this population, suggesting that post-menopausal women are potential targets for probiotic supplementation to increase bone mass density [81].

Taken together, these data suggest that, although the exact nature of the connection between probiotics, vitamins and minerals remain to be thoroughly characterized, they harbor a real therapeutic potential.

## Synergistic effects of probiotics, vitamins and minerals on human health

**Fig. 3:**
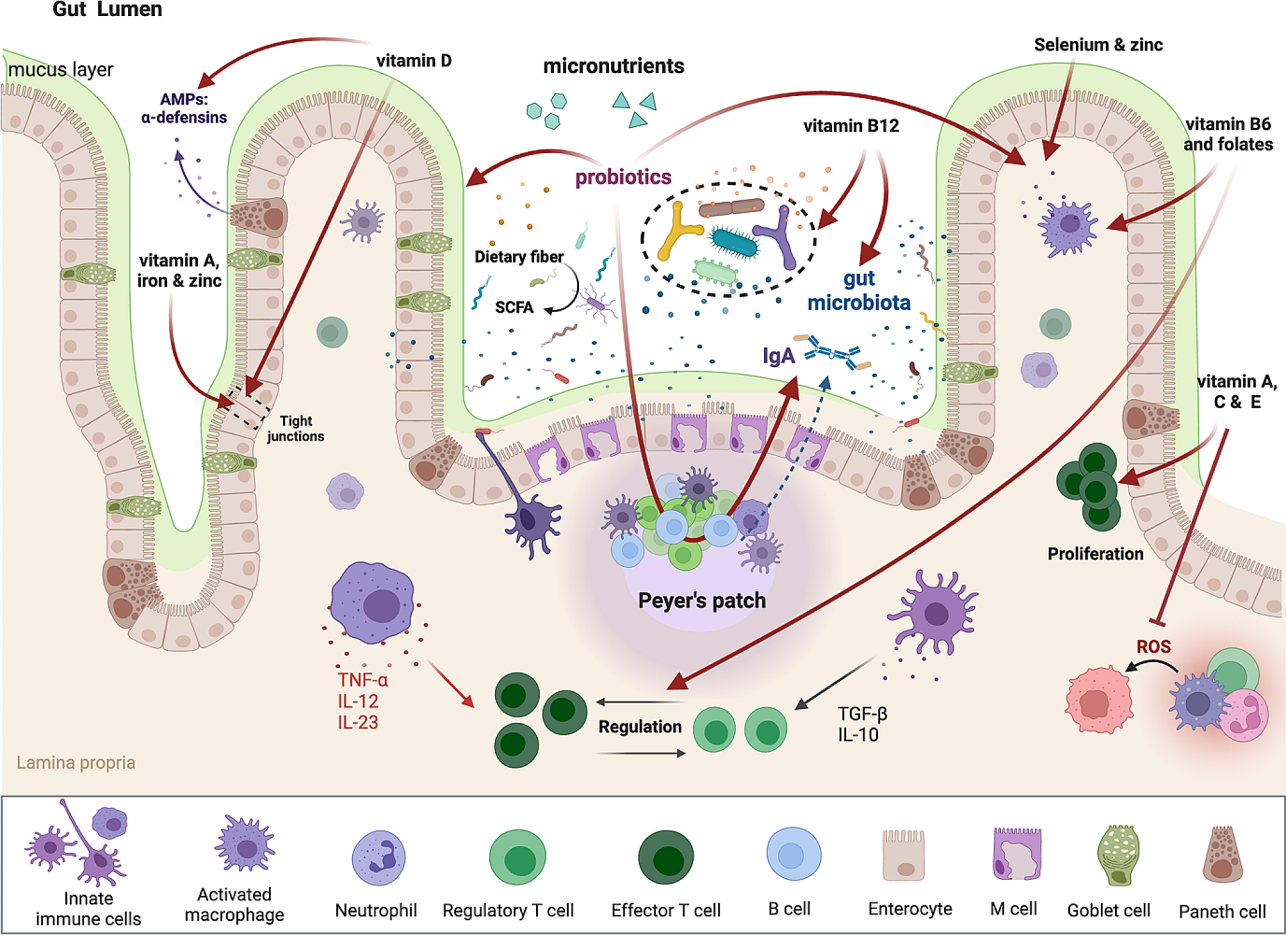
Proposed mechanisms by which probiotics act synergistically with micronutrients (vitamins and minerals) to stimulate intestinal immunity. This figure was created with Biorender.com

Accumulating evidence suggest that probiotics act synergistically with micronutrients (i.e. vitamins and minerals) to support intestinal immunity and promote human health [29]. It is now well established that the intestinal microbiota plays a major role in promoting and maintaining gut health [4, 10, 11, 19]. The available data suggest that, by restoring the composition of the human gut microbiota, probiotics exert multiple effects on host immunity [82]. Thus, interest in the immunomodulatory effects of probiotic bacteria and their therapeutic ability has increased considerably over the last decade [19, 83]. For example, probiotics improve intestinal health by competing with harmful pathogens for nutrients and attachment sites, reducing the growth of pathogens [84]. They produce antimicrobial substances such as lactic acid, hydrogen peroxide and antimicrobial peptides, which inhibit harmful bacteria [85, 86]. Probiotics also modulate immune responses by stimulating antibody production [87], and activating immune cells, which promotes a balanced immune system [84]. In addition, they improve intestinal barrier function by inducing mucus production [88] and regulating the expression of tight junction proteins [88], reducing permeability and preventing harmful bacteria and toxins from entering the bloodstream. All these are important actors for gut health and proper host-microbiota interaction and balance (Fig. 3).

Some studies highlighted the immunomodulatory potential of SCFAs through their action on G-protein coupled receptors, which coordinate signaling pathways and regulate gene expression in immune cells [89], as well as on intestinal T-cells, where SCFA-derived acetyl groups influence cytokine gene expression [90]. Based on these findings, a role has emerged for SCFAs as therapeutic targets in various autoimmune diseases [90]. Moreover, research on gut immunometabolism has demonstrated that probiotics can induce either pro-inflammatory [83] or anti-inflammatory [91] reactions in their host. Also, recent clinical studies indicated an effect of probiotics on both the innate and adaptive human immune responses, and modulation of antibody production after vaccination against influenza virus [92] or SARS-CoV-2 [93–96], for example.

Vitamins contribute to the normal functioning of the immune system by modulating the production of both pro-and anti-inflammatory molecules, supporting the epithelial barrier, and helping to balance and diversify the intestinal microbiota [97, 98]. Vitamins supplementation may directly impact gut immune system or it may act indirectly, through the production of microbial metabolites [98]. For example, vitamins A and D modulate the expression of tight junction proteins and stimulate mucosal immune responses [49] and the production of antimicrobial peptides [99, 100]; vitamin B6 and folates improve immune function [101]; vitamin B12 feeds the gut microbiota [102]; while vitamins C and E suppress the formation of reactive oxygen species [103]. There is growing evidence on the importance of the vitamin D pathway for intestinal homeostasis and signaling between gut microbiota and the host [104]. At higher concentrations, vitamin D favors the growth of protective microbiota, whereas low levels lead to a permeable barrier, outgrowth of harmful bacteria and increased inflammation [105].

Currently, fewer data are available on the role of minerals in gut health, but, similarly to vitamins, minerals appear to impact intestinal physiology (Fig. 3) [29, 106]. For instance, supplementation with zinc or iron was found to improve intestinal barrier function [107, 108], while zinc and selenium displayed immunomodulatory effects [109, 110]. Moreover, magnesium deficiency has been associated with the pro-inflammatory environment underlying the development of insulin resistance and type 2 diabetes, obesity and other metabolic conditions [111].

The interactions between the gut microbiota and micronutrients are bidirectional, i.e. micronutrients impact the composition and function of the microbiota, while the latter affects the bioavailability of vitamins and minerals [79]. Thus, while vitamins help regulate the microbiota-mediated production of metabolites, the reverse can also be true: bacterial strains such as LGG were shown to play a role in vitamin B homeostasis in the gut [52].

The effects of vitamins and probiotics supplementations on immune system have been explored in a range of pre-clinical and clinical studies. One such study, using in vivo and in vitro approaches, found that treatment with either retinol or retinoic acid inhibited norovirus replication by inducing changes in gut microbiota composition in mice [112]. A randomized controlled trial of 479 adult healthy volunteers reported that intake of a probiotic supplement containing *L. gasseri* PA 16/8, *B. longum* SP 07/3 and *B. bifidum* MF 20/5 along with vitamins and minerals for at least 3 months (including 2 winter/ spring periods) significantly reduced the incidence of respiratory tract infections *versus* placebo by 13.6%, shortened common cold episodes by almost 2 days and reduced symptom severity [113]. Meanwhile, iron supplementation in infants at risk of diarrhea and respiratory tract infections adversely affected the gut microbiota by lowering the abundance of bifidobacteria and lactobacilli and increasing the relative proportions of pathogens [114, 115].

Studies have also reported that probiotics and micronutrients can be effective in preventing fatigue and oxidative stress. For instance, in an observational cohort study of 242 subjects complaining of psychological stress (as defined by a Perceived Stress Scale score ≥ 21), stress and fatigue were significantly reduced by the intake of a food supplement containing *L. gasseri* PA 16/8, *B. bifidum* MF 20/5, *B. longum* SP 07/3, as well as vitamin A, B-group vitamins and magnesium; the effect was maintained one month after discontinuation [116]. Similarly, co-administration of vitamin D with a probiotic capsule containing *L. acidophilus, B. bifidum, L. reuteri* and *L. fermentum* over 12 weeks in 60 women with polycystic ovarian syndrome had beneficial effects on mental health (anxiety and depression) while significantly reducing levels of testosterone, high-sensitivity C-reactive protein, and malondialdehyde [117].

For instance, in a non-randomized clinical trial, participants who consumed a probiotic strain of *Lactiplantibacillus plantarum* 299v plus an iron supplement exhibited significantly greater iron absorption in the presence of the probiotic than without it [118].

A systematic review of the literature identified 14 studies with available data on the effect of probiotic supplementation on micronutrient status in healthy humans [29]. Published between 2000 and 2020, these studies were conducted in different geographical regions and had variable designs, with 11 being randomized [29]. While data on the impact of some probiotic strains, such as the symbiotic containing 4 different patented probiotic strains (*L. plantarum*, *L. acidophilus*, *B. infantis* and *B. lactis*, Hyperbiotics PRO-Kids: US patent 8,007,777 and 7,150,623) and a prebiotic (fructo-oligosaccharides, FOS), on the levels of fat-soluble vitamins (A and E) and carotenoids were inconclusive, a potential role emerged for selected strains (including *L. acidophilus* La1 [22] and *B. adolescentis* MB 239 [63]) in improving folate and vitamin B12 status. However, these positive results in healthy individuals need to be validated in larger clinical trials [29].

**Table 1:** summarizes some of the main probiotic strains currently known for their impact on gut health and when applicable their additional effects on the absorption and metabolism of vitamins, minerals and SCFA

Probiotic strain	Reported effects on intestinal health and host energy metabolism	Ref.
*Lactobacillus rhamnosus* GG	Improves intestinal barrier function, modulates immune responses, increases mineral absorption and stimulates butyrate-producing bacterial strains	[119–121]
*Bifidobacterium lactis* BB-12	Reduces intestinal inflammation, promotes immune function, B vitamins absorption and increases SCFA production	[122, 123]
*Lactobacillus* acidophilus La-5/ *L.* acidophilus DDS-1	Improves digestion and reduces symptoms of lactose intolerance, improves minerals absorption and increases SCFA production	[122–124]
*Bifidobacterium longum* BB536	Balances the intestinal microbiota and reduces gastrointestinal discomfort, improves calcium and iron absorption and increases the production of SCFA	[125, 126]
*Lactobacillus plantarum* 299v*/L. plantarum* 2362	Reduces intestinal inflammation, improves intestinal barrier function and improves absorption minerals	[127, 128]
*Bifidobacterium bifidum* Bf-688	Promotes the balance of intestinal microbiota and reduces inflammation, improves the absorption of vitamins and increases the production of SCFA	[129]
*Lactobacillus salivarius* UCC4331	Improves immune function and reduces IBS symptoms and SCFA production	[130]
VSL#3^1^ cockatil	Reduces intestinal inflammation and modulates the immune response and increases SCFA the production	[131, 132]

Table 1. Main effects on intestinal health and host energy metabolism of selected probiotic strains.

^1^VSL#3 = is a commercial probiotic mixture consisting of eight bacterial strains: 4 strains of *Lactobacillus* (*L. acidophilus*, *L. plantarum*, *L. casei*, and *L. delbrueckii* ssp. *bulgaricus*), 3 strains of *Bifidobacterium* (*B. breve*, *B. longum*, and B. *infantis*), and 1 strain of *Streptococcus* (*S. salivarius* ssp. *thermophilus*).

## Concluding remarks and future directions

Probiotic bacteria are promising players in host energy metabolism and gut health through the production of SCFAs, amino acids, vitamins, enzymes, and immunomodulatory compounds, some of which have been shown to display therapeutic potential. Moreover, many probiotics increase energy harvest by producing SCFAs and B-vitamins, which contribute to the cellular Krebs cycle.

Although there is increasing evidence describing the multiple effects of probiotics in the gut, more in vivo studies are needed to evaluate the ability of select probiotic strains to increase energy availability. Furthermore, current knowledges are mostly limited to the healthy general population and are not yet sufficient for formulating a convincing argument for regulatory bodies such as the European Food Safety Authority (EFSA).

Studies are also needed to improve the characterization of probiotics and their mode of action, starting with a single-strain strategy. Indeed, precision probiotics is an attractive alternative to the one-size-fits-all approach and could be used in the future for personalized intervention of probiotic strains to improve several pathologies based on the specific characteristics of each individual [133]. Future research is likely to be highly microbiota-driven; in particular, a promising niche of investigation will be to understand how different strains potentially drive vitamin production. Notably, although most probiotic strains are not able to produce B-vitamins, they could exert an effect on vitamin biosynthesis in the gut, which could be studied using a transcriptomic approach to determine the relative expression of genes involved in their metabolism.

Through their synergistic effects, probiotics and micronutrients play an important role in supporting intestinal immunity; however, the mechanisms involved are complex and still under investigation. The synergy between probiotics, vitamins and minerals is a promising area for further research, and well-designed clinical trials are needed to describe such interactions *in situ.* A better understanding of the interactions between probiotics, commensals and the host will drive the optimization of probiotic, vitamin and mineral supplementation in healthy individuals as well as in patients. To this end, a multidisciplinary approach involving microbiologists, physiologists, bioinformaticians and physicians appears warranted.

## Methodology

To appraise the existing data on the interplay between probiotics, vitamins and minerals in promoting gut health, three experts in the field were invited to present different aspects on this topic. Presentations were given during a one-day virtual meeting and each presentation was followed by extensive discussions within the panel. The information gathered and group consensus statements were incorporated into the present manuscript, together with a review of the relevant literature in the field, which was next thoroughly reviewed by the topic leaders.

We based our selection of discussed studies through PubMed search using probiotics, vitamins, minerals, micronutrients and clinical trial. This selection was thus performed as described in the ref # 41: *They first identified 2772 abstracts. Removing duplicates (n = 320) and screening studies by title and abstract, led to 22 human clinical trials and finally to 14 articles after elimination of articles that did not meet inclusion criteria.*

## Data Availability

No datasets were generated or analysed during the current study.
